# Estimating the Effects of Dental Caries and Its Restorative Treatment on Periodontal Inflammatory and Oxidative Status: A Short Controlled Longitudinal Study

**DOI:** 10.3389/fimmu.2021.716359

**Published:** 2021-09-15

**Authors:** Tatjana Kanjevac, Ervin Taso, Vladimir Stefanovic, Aleksandra Petkovic-Curcin, Gordana Supic, Dejan Markovic, Mirjana Djukic, Boris Djuran, Danilo Vojvodic, Anton Sculean, Mia Rakic

**Affiliations:** ^1^Department of Dentistry, Faculty of Medical Sciences, University of Kragujevac, Kragujevac, Serbia; ^2^Clinic for Stomatology, Military Medical Academy, Belgrade, Serbia; ^3^Institute for Medical Research, Military Medical Academy, Belgrade, Serbia; ^4^Department of Paediatric and Preventive Dentistry, School of Dental Medicine, University of Belgrade, Belgrade, Serbia; ^5^Department for Toxicology, Faculty of Pharmacy, University of Belgrade, Belgrade, Serbia; ^6^Faculty of Dental Medicine, University of Bern, Bern, Switzerland; ^7^ETEP (Etiology and Therapy of Periodontal Diseases) Research Group, Faculty of Dentistry, University Complutense of Madrid, Madrid, Spain

**Keywords:** caries, periodontium, periodontitis, dental restoration, cytokines, oxidative stress, restorative material, inflammation

## Abstract

Dental caries and periodontitis are among the most common health conditions that are currently recognized as growing socio-economic problems relating to their increasing prevalence, negative socio-economic impact, and harmful effects on systemic health. So far, the exact effects of caries and standard restorative materials on periodontal inflammatory and oxidative status are not established. The present study aimed to investigate the effect of caries and its restoration using standard temporary and permanent filling materials on a panel of 16 inflammatory and oxidative markers in gingival crevicular fluid (GCF) of periodontally healthy individuals, 7 (D7) and 30 (D30) days post-restoration, while the intact teeth represented the control. One hundred ninety systemically and periodontally healthy patients with occlusal caries underwent standard cavity preparation and restorations with one of six standard temporary or permanent restorative material according to indication and randomization scheme. Interleukin (IL)-2, IFN- γ, IL-12, IL-17A, IL-13, IL-9, IL-10, IL-6, IL-5, IL-4, IL-22, TNF-α, IL1- β, thiobarbituric acid reactive substances, superoxide dismutase, and reduced form of glutathione were measured in GCF samples by flowcytometry and spectrophotometry in aid of commercial diagnostic assays. Caries affected teeth exhibited significantly increased IL-1 β, IL-17, IL-22, and TBARS and decreased IL-9 concentrations compared to healthy controls. Treatment generally resulted in an increased antioxidant capacity with exception of zinc-polycarboxylate cement showing distinctive inflammatory pattern. Comparison of inflammatory and oxidative profiles in temporary and permanent restorations showed material-specific patterning which was particularly expressed in temporary materials plausibly related to greater caries extension. Caries affected teeth exhibited a balanced inflammatory pattern in GCF, with a general tendency of homeostatic re-establishment following treatment. Restorative materials did not provide specific pathological effects, although some material groups did exhibit significantly elevated levels of inflammatory and oxidative markers compared to healthy controls, while the material-specific patterning was observed as well.

## Introduction

Despite performant preventive strategies, caries and periodontitis are still amongst the most prevalent infectious disease of mankind, which mostly relates to the decreased rate of tooth loss and prolonged human lifespan (1–3). These pathologies are currently considered as a major public health problems related to a substantially negative impact in overall health and oral-health-related-quality of life (OHRQoL) (4–6), also representing a major financial burden in oral health care (2). Caries and periodontitis share a similar pathogenetical pattern of chronic inflammation induced by dysbiotic biofilms (5, 7, 8), and unrestricted caries progression leading to apicalization of pathological process with further development of endo-periodontal lesions (9). Paradoxally, the nature of caries-periodontium interaction in health and disease is still elusive and yet to be established. Thus, the 12th European Workshop on Periodontology jointly conducted by European Federation of Periodontology (EFP) and European Organization for Caries Research (ORCA) was dedicated to this important concern (10). The recent research studies reveal that activation of the circumpulpal odontoblasts network followed by release of cytokines and recruitment of the immunological cells in dental pulp occur already in the early-stage enamel caries (11). Considering the tight topographic communication between endodontium and periodontium, there is an increased risk of pro-inflammatory effects of caries and its treatment on periodontium. Immunopathological pattern in caries and periodontitis implies the activation of the nuclear factor kappa B (NF-kB) in response to the bacterial challenge, followed by biosynthesis of pro-inflammatory cytokines and elicitation of T-lymphocyte helper (Th)-1 pro-inflammatory response (12, 13). The unrestricted disease progression in both pathologies results in activation of Th-17 response mediated by M-1 macrophages (9). This is deemed important since the shared immunological pattern allows for synergistic pro-inflammatory effects among diseases, as recently confirmed based on significantly higher levels of IFN-γ, IL-1β, IL-2, IL-4, and IL-6 in the gingival crevicular fluid (GCF) of caries affected periodontally healthy teeth compared to intact teeth (14). Moreover, it is established that some components of standard restorative materials may cause pro-inflammatory effects coupled with depletion of antioxidants and increase in reactive oxygen species (ROS) even at non-cytotoxic concentrations (15–17). Additionally, restorative materials may interfere with local immunological networks and alter the sensing between Toll-like receptors (TLRs) and lipopolysaccharides (LPSs) (18). Hence, the characterization of biological interaction between commonly used restorative biomaterials and periodontium is of critical importance since the preconditions for their respective pathological interplay and additive inflammatory effects in state of periodontal disease undoubtfully exist related to shared immunopathological patterns. Finally, the continual assessment of commercial restorative biomaterials in the clinical practice represents a backbone of their safe and effective use (19, 20). Several experimental studies have demonstrated that restorative materials may alter biological local response, while the intensity and final outcomes greatly varied amongst different biomaterials (21–24). Scarce few clinical studies reported on the effects of standard restorative materials on periodontal markers, demonstrating the association of restorative treatment with significant increase in Th-1 and Th-17 markers 7, 14, 21, and 30 days post-treatment (14, 25, 26). However, those very initial studies were conducted in relatively small sample and without comprehensive assessment of various materials and biomarkers, thus the knowledge about specific biological effects of commonly used restorative materials on periodontal status remains scarce.

The knowledge about the effects of caries on periodontal homeostasis remains of great importance also in context of possibly negative effects on systemic conditions, since periodontitis and caries represent “silent” inflammatory burdens that may negatively affects systemic diseases and efficacy of respective therapies (27). In brief, the specific histomorphology of periodontal tissues and local vasculature contribute to fast decompartmentalization of the periodontal inflammation *via* haematogenous dissemination of periodontal bacteria and/or inflammatory mediators *via* bloodstream, which may affect progression and treatment responsiveness in inflammation-driven pathologies such as cardio-metabolic, neurodegenerative, autoimmune diseases, and cancer (28). So far, periodontal inflammation is positively correlated with diabetes mellitus, adverse pregnancy outcomes, cardiovascular diseases, rheumatoid arthritis, and Alzheimer’s disease, while it has been demonstrated that periodontal treatment improves surrogate markers of comorbid conditions (27–29). Given the fact that 91% of adults (age of 20–64 years) are affected by caries, establishing the exact inflammatory profile underlying caries-periodontal interaction remains of great importance.

The working hypothesis was that carries and its treatment upregulated inflammatory and oxidative markers in periodontal tissues.

Objective of the study was to estimate the effects of dental caries and its restorative treatment using standard temporary and permanent dental filling materials on periodontal inflammatory and oxidative status in periodontally healthy individuals 7 and 30 days post-restoration.

## Materials and Methods

**Figure 1:**
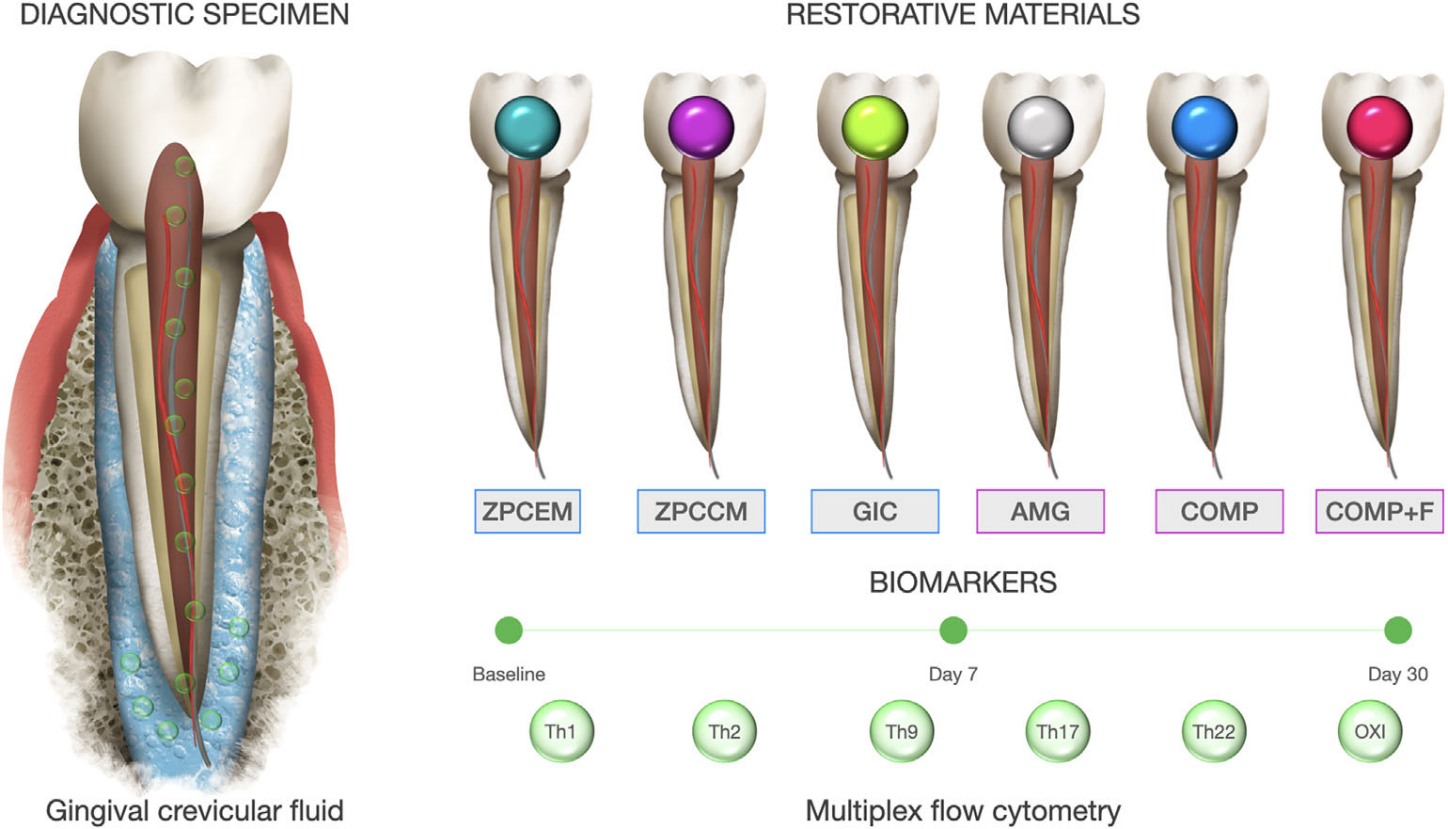
Graphical flowchart and study design. Superoxide dismutase (SOD), expressed as U SOD/mg proteins. 3) Reduced form of glutathione (GSH).

This study was designed as a short longitudinal controlled study assessing the GCF levels of 13 inflammatory markers of T-helper (Th) response including Th1, Th2, Th9, Th17, and Th22, and 3 oxidative markers baseline, 7 (D7) and 30 (D30) days after routine caries restorative treatment with commonly used commercial filling materials (**Figure 1**). Control group (HC) consisted of GCF samples from intact healthy teeth at nonadjacent position from the same morphological group.

### Study Population and Criteria

One hundred ninety patients attending the Clinic for Stomatology, Military Medical Academy, Republic of Serbia from the October 2018 until December 2020 were enrolled in the study. All participants were informed on the study and agreed to participate by signing an inform consent. Study fully adhered to the Declaration of Helsinki of 2008 and was approved by the Institutional Ethical Committee (Ethical Committee of the Military Medical Academy, Ministry of Defense, Serbia). Participants were included if being systemically healthy non-smokers with clinically healthy periodontium (30, 31), if having at least one lateral tooth affected by occlusal caries and one healthy contralateral tooth from the same group. Exclusion criteria were as follows: 1) periodontal treatment in the preceding year; 2) intake of antibiotics in the preceding 6 months; 3) intake of anti-inflammatory drugs in preceding month; 4) pregnant or lactating females; and 5) deep caries lesions that do not allow complete caries removal in the first session. In case of presence of multiple caries lesions, the teeth that were not subject of the study were consecutively treated in upcoming sessions as well.

### Restorative Materials and Experimental Groups

Caries lesions were diagnosed using standard visual-tactile examinations, in some cases coupled with bitewing radiograph to confirm the presence of interproximal caries (32). Tooth with the worst caries destruction was defined as representative sampling site in case of multiple lesions, while the criterion for control was accessibility. Cavity preparations were performed by two experienced dentists (ET and VS) according to the standard principles for adhesive cavity or conventional Black cavity principles (33) and filled with materials according to indication for temporary or permanent restoration. The clinical criterion for application of temporary restorative material was presence of expressed hypersensitivity in caries lesions with radiologically clear demarcation between carious destruction and pulpal chamber. Two randomisation schemas were built, one for permanent and one for temporary restorations in aid of home-made customizable software that was set to ascertain equal but random material allocation, as well as allocation of amalgams in molar region as follows:

*Temporary restorative materials:*

1. ZPCEM: Zinc-phosphate cement (Cegal NV, Galenika, R Serbia)
2. ZPCCEM: Zinc-polycarboxylate cement (Harvard, USA);
3. GIC: Glass Ionomer cement with fluoride release (GC Fuji PLUS^®^, Green Circle, USA)

*Permanent restorative materials:*

1. AMG: Amalgame (Extracap D caps, Galenika, R Serbia);
2. COMP: Nanohybrid composite -the mixture of 2.5–10% of bisphenol- A-diglycidyl-dimethacrylate (BisGMA) and 2.5–10% of urethane-dimethacrylate (UEDMA) and non-hazardous additions (Tetric EvoCeram,Ivoclar Vivadent, USA);
3. COMP+F: Nanohybrid composite with fluoride release and recharge-the mixture of BisGMA 15–25%, triethyleneglycol- dimethacrylate (TEGDMA) 12–14%, aluminofluoroborosilicate glass 50–60%, aluminium trioxide (Al2O3) 1–2%, and DL-Camphorquinone (Beautifill II, Shofu Inc., Japan)

The investigators implicated in laboratorial and data analysis have been blinded for specimen affiliation, which was ascertained by specific number encryption of the specimen generated for each tooth in the excel sheet by established schema and handled by an in charged investigator (BDJ). The same investigator performed specimens deciphering following laboratorial analyses and final database preparation for statistical analyses.

In case of resins, the one step self-etch agent was applied and polymerized according to manufacturer instruction (G-Bond, GC, Tokyo, Japan), while for amalgam restorations was used ZPCEM. Biomaterials were inserted in small portions using horizontally layer technique for composite materials and standard condensation technique for AMG, they were anatomically formed using standard hand instruments, surface adjustment was performed using hand tools for AMG, while the finishing and polishing phases were performed using diamond finishing burs and polishing discs. The multi-stage light polymerization was performed by emission of 1000 mW/cm^2^ for 20 s per each layer and 40 s for last layer was performed for composite materials, while the curing time for GIC was 6 min. The material portion per defect ranged from 0.07 to 2.03 g.

### Measurement of Biomarkers

GCF samples were collected, processed, and stored according to previously reported protocol (34). In brief, sample collection was performed 24 h following clinical examination to avoid possible contamination with blood; however, the strips visually contaminated with blood or saliva were discarded. Samples were collected baseline for all groups, as well as D7 and D30 in experimental groups. Sampling site was air dried and isolated with cotton rolls, the supragingival plaque was removed, and following that the fine sterile paper strips (PerioPaper, ProFlow, Amityville, NY) were inserted into the peri-implant sulcus until mild resistance and left for 30 s according to sampling time method (35). Paper strips were further inserted into microcentrifuge plastic tubes containing 0.5 ml sterile phosphate-buffered saline and transported to the laboratory. Samples were vortexed for 10 s and further centrifuged for 5 min at 3,000g in order to separate the cells and debris, and then the paper strips were removed. The obtained samples were stored at -20°C biochemical analyses.

Inflammatory markers were measured using flowcytometric method in aid of commercial diagnostic assay (Flowcytomix, Human Th Cytokine Panel 13-plex, Cat No 740001, LEGEND plexTM, Biolegend, San Diego, CA 92121, USA) with the following detection limits: IL-2 (1.0 pg/ml), IFN- γ (1.0 pg/ml), IL-12 (1.1 pg/ml), IL-17A (1.5 pg/ml), IL-13 (1.4 pg/ml), IL-9 (1.9 pg/ml), IL-10 (1.1 pg/ml), IL-6 (1.1 pg/ml), IL-5 (1.1 pg/ml), IL-4 (0.7 pg/ml), IL-22 (2.0 pg/ml), TNF-α (1.0 pg/ml), and IL-1β (1.0 pg/ml).

Oxidative markers were estimated spectrophotometrically according to previously reported protocol (36) as follows:

1. Malondialdehyde (MDA) was measured by thiobarbituric acid reactive substances (TBARS) production method, expressed as nmol MDA/mg proteins
2. Superoxide dismutase (SOD), expressed as U SOD/mg proteins
3. Reduced form of glutathione (GSH) was measured using enzymatic recycling assay, expressed as nmol TNB/mg proteins

Total protein concentrations were estimated in GCF supernatants according to Lowry et al. (37).

### Statistical Analysis

The primary outcome variables were biomarker concentration in GCF samples from caries affected and intact teeth, and their respective changes in response to different restorative materials over the time. Secondary outcome variables were difference in biomarker concentrations between caries (pooled baseline values) and HC. In lack of referent diagnostic ranges for measured biomarkers, the sample size calculation was performed based on IL-17 changes following caries treatment established in parallel study conducted by this research group. Considering estimated standard deviation of 1.94 as a preferred difference before and following treatment with 90% of power, the estimation resulted in 28 teeth per group, but the sample was preventively increased to limit potential attrition bias. The normality of outcome variables was tested using Shapiro–Wilk test. Biomarkers between the groups were compared using Man-Whitney U test, while the values were expressed as mean concentration and standard deviation being the tooth the unit of analysis. The changes in biochemical markers between follow-ups were estimated using Wilcoxon signed-rank test for paired samples. Data analyses were performed using commercial software (Prism 5.0, GraphPad Software, Inc., La Jolla, CA, USA).

## Results

Total sample included 190 patients, while 178 patients completed the study follow-up, the attrition was mostly observed in group of temporary fillings (ZPCCEM:3; ZPCEM: 4; GIC:2) reason with loss of restoration as a most frequent cause, while in group of permanent restorations only two patients missed the D30 follow-up, which did not affect study outcomes according to sample size estimation. Final sample comprised 87 females and 91 males, with average age of 28 years (range: 18–34 years) with comparable distribution of both gender and age amongst the groups (**Table 1**).

**Table 1:** Characteristics of the study population.

Characteristics	Temporary restorations			Permanent restorations		
	ZPCEM	ZPCCEM	GIC	AMG	COMP	COMP+F
	(n = 34)	(n = 33)	(n = 33)	(n = 30)	(n = 30)	(n = 30)
Gender						
*Male*	16	17	15	16	15	17
*Female*	18	16	18	14	15	13
Mean age	28.1 ± 6.12	26.7 ± 4.23	25.5 ± 3.73	28.6 ± 4.13	26.2 ± 5.21	24.8 ± 4.71
Number of teeth (n, mean and range)	27.24 (26–28)	27.87 (25–28)	27.55 27–28)	26.34 (27–28)	27.65 25–28)	26.67 (26–28)

ZPCEM, zinc-phosphate cement; ZPCCEM, zinc-polycarboxylate cement; GIC, glass ionomer cement with fluoride release; AMG, amalgam; COMP, nanohybrid composite; COMP+F, nanohybrid composite with fluoride release and recharge.

### Biomarker Changes 7 and 30 Days Post-Restoration

**Figure 2:**
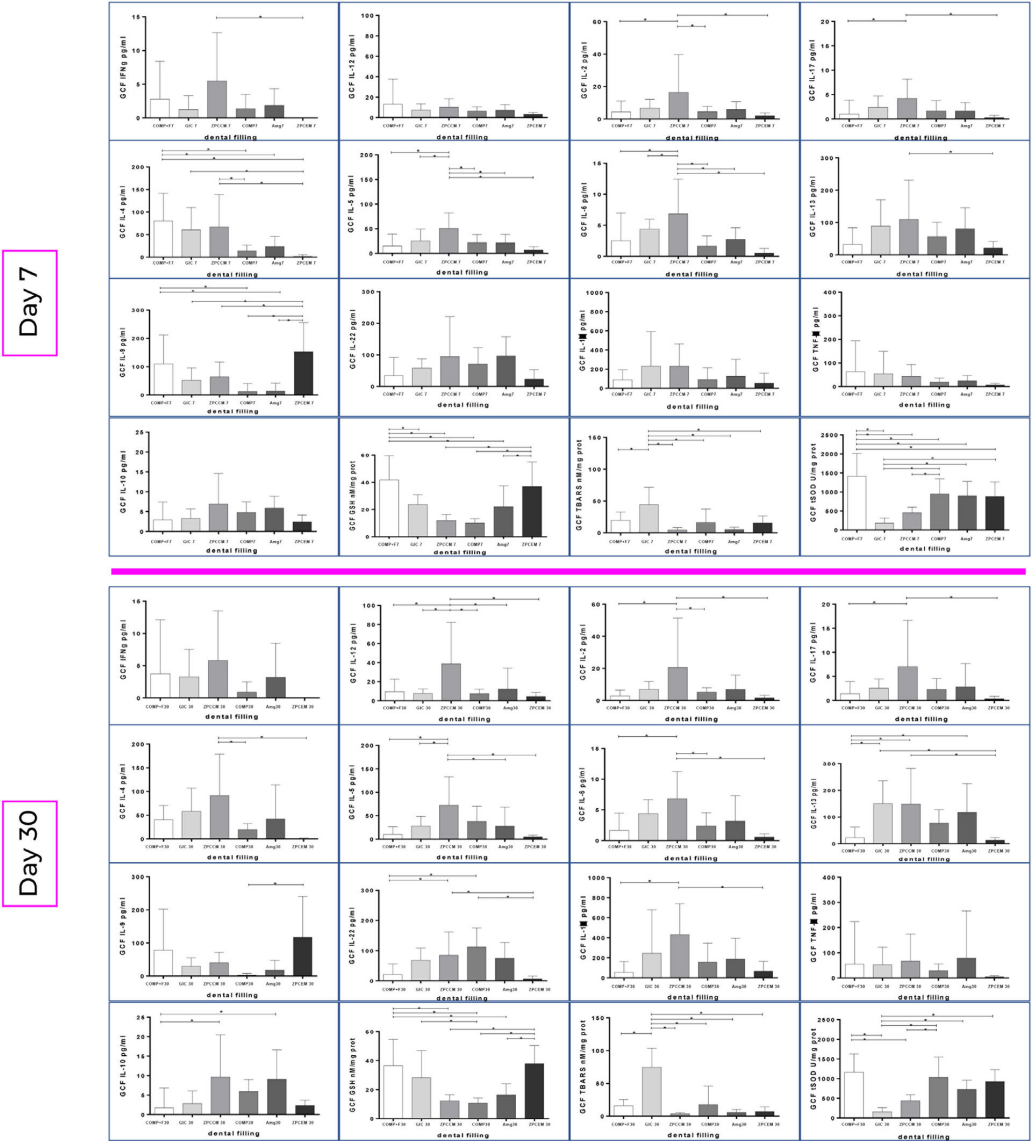
Biomarker changes from baseline to 7 and 30 days post treatments by the experimental groups. ZPCEM showed significant changes at the 30D; thus, the levels of IL-17 significantly declined (p = 0.037), while reduced form of glutathione (GSH) and superoxide dismutase (SOD) increased compared to baseline (p < 0.05). In GIC, the GSH significantly increased from baseline to 7D (p = 0.036) and 30D (p = 0.021). In ZPCCEM, the levels of Th1 and Th2 markers as well as SOD significantly increased D7, while D30 in addition to Th1 and Th2 markers the IL-17 significantly increased relative to baseline (p < 0.05). In GIC group, GSH was single marker that significantly increased D7 (p = 0.045) and D30 (p = 0.015) compared to baseline. In AMG, the levels of IL-12 (p-0.30) and IL-22 (p = 0.027) significantly declined D30, while GSH (p = 0.040) and SOD (p = 0.030) levels significantly increased D7 and D30. In COMP, the levels of IL-13 (p = 0.045) and IL-22 (p = 0.021) significantly decreased D7, D30 IL-5 declined when compared to baseline (p = 0.027), while IL-17 and SOD significantly decreased at D7 and D30 (p < 0.05). In COMP+F, the levels of IL-17 declined (p = 0.045), while TNFα, GSH, and SOD significantly increased D7 (p < 0.05), and D30 IL-17, GSH and SOD preserved the trend from D7 (p < 0.05), while IL-5 declined compared to baseline (p = 0.040). ZPCEM, zinc phosphate cement; GIC, glass ionomer cement; ZPCCM, zinc-polycarboxylate cement; AMG, amalgam; COMP-resin, COMP+F-fluoride loaded resin; *p < 0.05.

The changes in biomarker levels in response to treatment and over the time and by different restorative materials are portrayed in **Figure 2**. ZPCEM did not show significant changes between baseline and 7D, while at the 30D the levels of IL-17 significantly declined (p = 0.037) and GSH and t-SOD increased compared to baseline (p < 0.05). In GIC, the GSH showed tendency of continual increase from baseline till 7D (p = 0.036) and 30D (p = 0.021). In ZPCCEM, the levels of Th1 and Th2 markers as well as SOD significantly increased D7, while D30 in addition to Th1 and Th2 markers the IL-17 significantly increased relative to baseline (p < 0.05). In GIC group, GSH was single marker that significantly increased D7 (p = 0.045) and D30 (p = 0.015) compared to baseline. In AMG, the levels of IL-12 (p-0.30) and IL-22 (p = 0.027) significantly declined D30, while GSH (p = 0.040) and SOD (p = 0.030) levels significantly increased D7 and D30. In COMP, the levels of IL-13 (p = 0.045) and IL-22 (p = 0.021) significantly decreased D7, D30 IL-5 declined when compared to baseline (p = 0.027), while IL-17 and SOD significantly decreased at D7 and D30 (p < 0.05). In COMP+F, the levels of IL-17 declined (p = 0.045), while TNFα, GSH, and SOD significantly increased D7 (p < 0.05), and D30 IL-17, GSH, and SOD preserved the trend from D7 (p < 0.05) and IL-5 additionally declined compared to baseline (p = 0.040).

### Biomarkers Levels Between Different Restorative Materials

**Figure 3:**
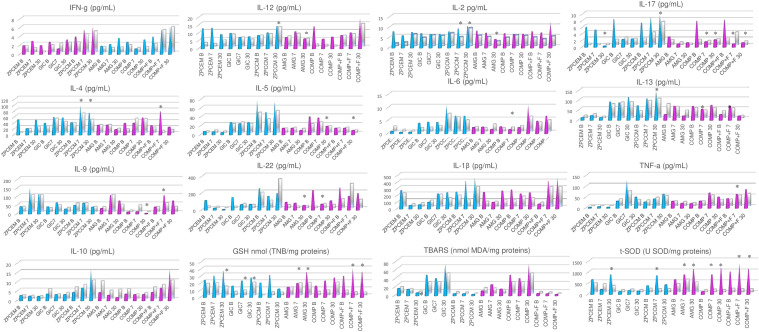
Biomarker levels between different restorative materials 7 and 30 days post-restoration. ZPCCM exhibited remarkably increased values of Th1 (IFNγ and IL-2; p < 0.05), Th2 (IL-4, IL-5, Il-6 and IL-13 p < 0.05), Th13 (IL-13; p = 0.045), and Th17 (IL-17; p = 0.021) markers. ZPCCM and COMP+F exhibited significantly elevated levels of IL-4 that were significantly higher compared to ZPCEM and COMP (p < 0.05), and for all other permanent fillings, respectively. The highest GSH levels were demonstrated for reduced form of glutathione (GSH) in ZPCEM and COMP+F, for superoxide dismutase (SOD) in COMP and COMP+F, and for thiobarbituric acid reactive substances (TBARS) in GIC that simultaneously demonstrated significantly reduced SOD values compared to all permanent fillings and ZPCEM (p < 0.05). D30 ZPCCM conserved significantly increased Th1(IL-1 and IL-2; p < 0.05), Th2 (Il-4, Il-5, IL-6; p < 0.05), Th13 (IL-13; p = 0.040), and Th17 (IL-17; p = 0.037) markers, while IL-22 was additionally increased in this timepoint (p = 0.021). Regarding inflammatory markers, GIC, ZPCEM, and AMG showed increased values of IL-13, IL-9, and IL-10, respectively (p < 0.05). ZPCEM, GIC, and COMP+F exhibited the highest GSH values (p < 0.05), GIC maintained the trend of the highest TBARS values (p = 0.001), while COMP and COMP+F exhibited the highest SOD values (p < 0.05). ZPCEM, zinc phosphate cement; GIC, glass ionomer cement; ZPCCM, zinc-polycarboxylate cement; AMG, amalgam; COMP-resin, COMP+F-fluoride loaded resin; *p < 0.05.

The biomarker levels between different restorative materials are depicted in **Figure 3**. The most distinctive profile regarding inflammatory markers was demonstrated in ZPCCM that exhibited remarkably increased values of Th1 (IFNγ and IL-2; p < 0.05), Th2 (IL-4, IL-5, IL-6, and IL-13 p < 0.05), Th13 (IL-13; p = 0.045), and Th17 (IL-17; p = 0.021) markers. ZPCCM and COMP+F exhibited significantly elevated levels of IL-4 that were significantly higher compared to ZPCEM and COMP (p < 0.05), and for all other permanent fillings, respectively. Regarding oxidative markers, the highest GSH levels were demonstrated for GSH in ZPCEM and COMP+F, for SOD in COMP and COMP+F, and for TBARS in GIC that simultaneously demonstrated significantly reduced SOD values compared to all permanent fillings and ZPCEM (p < 0.05).

D30 ZPCCM conserved the trend from D7 of remarkably increased Th1 (IL-1 and IL-2; p < 0.05), Th2 (Il-4, Il-5, IL-6; p < 0.05), Th13 (IL-13; p = 0.040), and Th17 (IL-17; p = 0.037) markers, while IL-22 was additionally increased in this timepoint (p = 0.021). Regarding inflammatory markers, GIC, ZPCEM, and AMG showed increased values of IL-13, IL-9, and IL-10, respectively (p < 0.05). Regarding oxidative markers, ZPCEM, GIC, and COMP+F exhibited the highest GSH values (p < 0.05), GIC maintained the trend of the highest TBARS values (p = 0.001), while COMP and COMP+F exhibited the highest SOD values (p < 0.05).

### Biomarker Levels Between Experimental Groups and Healthy Controls

The comparison of biomarker levels between healthy controls and baseline, D7, and D30 values around different restorative materials are listed in **Table 2**. Baseline values of all experimental groups have shown comparable values with exception of TNFα that was lower in ZPCCEM (p = 0.047). The baseline values were thus pooled into CARIES group and showed significantly increased levels of Th1 (IL-1 β), Th17 (IL-17), Th22 (IL-22), and free radical (TBARS), as well as decreased IL-9 when compared to HC (p < 0.05). In ZPCEM, the D7 values of IL-2, IL-4, and t-SOD were significantly higher than HC (p < 0.05), while D30 values of IFNγ (p = 0.040) remained significantly increased, and GSH (p = 0.049) significantly lower compared to HC. GIC showed significantly decreased IL-9 D7, while TBARS was significantly higher both D7 and D30 relative to HC (p < 0.05). In ZPCCEM, SOD was significantly higher D7, while IFNγ was significantly higher and GSH was significantly lower D7 and D30 compared to HC (p < 0.05). In AMG, SOD was significantly increased D7 and D30, while IL-10 was significantly decreased D30 (p < 0.05). COMP group exhibited significantly increased IL-4 D7 and increased IL-2 D30, while IL-9 was decreased D7 and D30 compared to HC. COMP+F group exerted significantly increased GSH and SOD D7, while IL-2 and IFNγ were significantly higher both D7 and D30 compared to HC (p < 0.05).

**Table 2:** Biomarker levels between experimental groups and healthy controls in different timepoints.

	HC	C	ZPCEM		GIC		ZPCCEM		AMG		COMP		COMP+F	
			7	30	7	30	7	30	7	30	7	30	7	30
**IL2**	5.215 (5.275)	**NS**	▲*	**NS**	**NS**	**NS**	**NS**	**NS**	**NS**	**NS**	**NS**	▲*	▲*	▲*
**IL12**	4.056 (49.727	**NS**	**NS**	**NS**	**NS**	**NS**	**NS**	**NS**	**NS**	**NS**	**NS**	**NS**	**NS**	**NS**
**IFNγ**	1.136 (3.325)	**NS**	**NS**	▲*	**NS**	**NS**	▲*	▲*	**NS**	**NS**	**NS**	**NS**	▲*	▲*
**IL17**	0.977 (2.795)	▲*	**NS**	**NS**	**NS**	**NS**	**NS**	**NS**	**NS**	**NS**	**NS**	**NS**	**NS**	**NS**
**IL4**	27.568 (47.053)	**NS**	▲*	**NS**	**NS**	**NS**	**NS**	**NS**	**NS**	**NS**	▲*	**NS**	**NS**	**NS**
**IL5**	27.863 (45.622)	**NS**	**NS**	**NS**	**NS**	**NS**	**NS**	**NS**	**NS**	**NS**	**NS**	**NS**	**NS**	**NS**
**IL6**	2.659 (10.063)	**NS**	**NS**	**NS**	**NS**	**NS**	**NS**	**NS**	**NS**	**NS**	**NS**	**NS**	**NS**	**NS**
**IL10**	3.886 (4.265)	**NS**	**NS**	**NS**	**NS**	**NS**	**NS**	**NS**	**NS**	▼*	**NS**	**NS**	**NS**	**NS**
**IL-13**	60.988 72.826	**NS**	**NS**	**NS**	**NS**	**NS**	**NS**	**NS**	**NS**	**NS**	**NS**	**NS**	**NS**	**NS**
**IL-9**	65.136 (102.424)	▼*	**NS**	**NS**	▼*	**NS**	**NS**	**NS**	**NS**	**NS**	▼*	▼*	**NS**	**NS**
**IL-22**	10.590 (24.571)	▲*	**NS**	**NS**	**NS**	**NS**	**NS**	**NS**	**NS**	**NS**	**NS**	**NS**	**NS**	**NS**
**IL-1β**	56.341 (243.941)	▲*	**NS**	**NS**	**NS**	**NS**	**NS**	**NS**	**NS**	**NS**	**NS**	**NS**	**NS**	**NS**
**TNFα**	3.852 (4.638)	**NS**	**NS**	**NS**	**NS**	**NS**	**NS**	**NS**	**NS**	**NS**	**NS**	**NS**	**NS**	**NS**
**GSH**	21.951 (15.048)	**NS**	**NS**	▼*	**NS**	**NS**	▼*	▼*	**NS**	**NS**	**NS**	**NS**	▲*	**NS**
**TBARS**	5.324 (36.389)	▲*	**NS**	**NS**	▲*	▲*	**NS**	**NS**	**NS**	**NS**	**NS**	**NS**	**NS**	**NS**
**t-SOD**	746.789 (479.659)	**NS**	▲*	**NS**	**NS**	**NS**	▼*	**NS**	▲*	▲*	**NS**	**NS**	▲*	**NS**

HC, intact healthy teeth; C, pooled baseline values from all experimental groups; ZPCEM, zinc phosphate cement; GIC, glass ionomer cement; ZPCCM, zinc-polycarboxylate cement; AMG, amalgam; COMP-resin, COMP+F-fluoride loaded resin; the biomarker values in the HC group are expressed as mean and standard deviation; ▲*: higher than control p < 0.05, ▼*: lower than control p < 0.05; NS, not significant p > 0.05.

## Discussion

Results of the present study show that carries, its treatment, and related commonly used restorative materials affect periodontal inflammatory and oxidative parameters. Caries affected teeth exhibited significantly increased proinflammatory markers in GCF compared to healthy intact teeth, while the treatment primary resulted in an improved antioxidant capacity. Overall, caries was associated with balanced inflammatory pattern in GCF without specific pathological characteristics, with general tendency of homeostatic re-establishment following treatment, while standard restorative materials generally did not exhibit harmful effects on periodontal markers. However, in some groups, inflammatory and oxidative markers remained significantly increased following treatment compared to HC, and material-specific patterning has been observed.

The immunopathological pattern shared between caries and periodontitis provides the basis for their pathological interplay due to a tight topographic communication of respective tissues. Caries affected teeth exhibited significantly increased pro-inflammatory markers of Th-1, Th-17, and Th-22 lymphocytes subgroups, as well as significantly increased marker of oxidative stress which is concordant to the previously reported finings (14, 26, 36). Implication of Th-17 response indicates an advanced inflammation grade, since IL-17 plays as inflammatory enhancer in pro-longed or unsuccessful elimination of detrimental noxa by Th-1 response, which is additionally supported with findings of an increased oxidative stress (TBARS) and depletion of protective Th-9 in caries. In context of periodontal tissues, although the inflammatory response was balanced based on coupled pro- and anti-inflammatory response, these findings clearly demonstrate that caries might provide additive inflammatory effects in periodontal disease. Additionally, IL-17 may specifically aggravate alveolar bone resorption since this mediator remains to be one of the key bone cytokines implicated in inflammatory osteoclastogenesis (38–40). Interestingly, IL-17 was the most treatment-affected cytokine since ZPCEM, COMP, and COMP+F caries restorations resulted in a significant decrease of this important bone cytokine. The clinical implication of this finding is importance of timely caries to prevent potentially deteriorating effects in alveolar bone resorption that apparently may occur even in stage of initial caries lesions. Caries treatment generally resulted in increased antioxidant capacity and decreased pro-inflammatory markers, except for ZPCCM that exerted the most distinctive inflammatory profile. The ZPCCM displayed a remarkably increased Th-1, Th-2, and Th17 response over time and compared to other materials. In fact, the increased pro-inflammatory markers in ZPCEM and ZPCCM that remained significantly higher compared to HC even at D30. Such finding might be related to larger caries extension present in teeth clinically indicated for temporary restorations, since the remaining dentine thickness is considered as a critical regulator of the pulpal response (41); thus, the stronger odontoblast stimulation possibly resulted in increased released of pro-inflammatory mediators in these groups. Additionally, the increased permeability of dental tubules may facilitate acid perfusion, resulting in a reactive inflammatory response of the odontoblasts and dental pulp.

Biocompatibility of dental materials became an important issue in dentistry, as the harnessing of new highly sensitive biomedical methods showed that elution of biomaterials when exposed to aggressive oral environment may cause serious adverse reactions (20). Out of these reasons, post-market clinical monitoring of dental materials by means of clinical and laboratory studies are subject of strongest recommendation (42) for securing the material safety and identification of potentially harmful components as a targets for material improvement (20, 43). To the best of our knowledge, this is the first study to report the effects of commonly used temporary and permanent dental filling materials on a wide panel of inflammatory and oxidative markers in GCF. The residual monomers and metal ions from resin restorations and amalgams such as HEMA and TEGDMA monomers, Hg^+2^ and Ni^+2^ are undoubtfully in major focus of biocompatibility concerns since these components may cause oxidative stress and chronic inflammation even at non-cytotoxic concentrations (18). Biological reaction on restorative biomaterials usually implies a time-limited adaptive inflammatory response with clinical signs of inflammation in adjacent dental pulp and gingiva (20), while at the molecular level it causes increased Th1, Th17, and Th22 marker in GCF samples (14, 26). Present study has demonstrated that restorative materials alter a periodontal inflammatory and oxidative status over the time, generally characterized with non-specific balanced inflammatory response and increased antioxidants, suggesting non-pathological inflammatory effects of commonly used restorative materials, which is in accordance to previously reported findings (26). The harmful effects of amalgam fillings characterized with oxidative damage due to mercury leach and subsequent reaction with thiol and/or selenol groups from endogenous molecules were probably one of the major concerns regarding dental material biocompatibility (44). In the present study, amalgam did not provide a significantly different inflammatory or oxidative profile in GCF when compared to the other materials, confirming no direct adverse effects of amalgam on periodontal homeostasis.

Furthermore, the antimicrobial components that are highly performant for bacterial growth control under composites and on the gingival margin, may also seriously interfere with material biocompatibility and contribute to the pro-inflammatory effects (20, 45), which is why this study was specifically designed to include one temporary and one permanent fluoride-containing restorative material. In the present study, the fluoride-loaded temporary and permanent materials did not provide significantly distinct inflammatory and oxidative effects on periodontium compared to other materials, with exception of GIC exhibiting significantly increased TBARS levels compared to other materials at both D7 and D30. Although the fluorides exert the capacity to induce oxidative stress (46), it should be also considered that GIC group showed distinctively higher baseline levels which might be indicative of a more extensive carious destruction rather than the specific biomaterial effect. Regarding oxidative parameters, other materials were generally associated with increased antioxidants while the GSH depletion was not observed following treatment, suggesting that dental restorations do not contribute to the periodontal oxidative stress. The general trend of increased antioxidants D7 post-restoration observed amongst the groups as well as compared to HC is suggestive of some transitory stimulation of antioxidative defenses but with no specific pathological impacts.

Regarding the effects of restorative materials on periodontal status, the continual clinical monitoring of biomaterials by means of highly predictive clinical and *In vitro* diagnostics (IVDs) remains of paramount importance for safe and effective use of biomaterials. Overall results suggest that conventional restorative materials do not provide direct pathological effects in periodontally healthy individuals; however, the observed material-specific patterning suggests cautious use of biomaterials, since apparently, they have potential to alter inflammatory and oxidative periodontal status, causing a low-grade inflammation that may act as an inflammatory enhancer in periodontal disease. The systemic effects of caries should be cautiously considered as well in context of facilitated hematogenous dissemination *via* periodontal vasculature, particularly in state of periodontal disease when the vascular permeability remains proportionally increased (47). In brief, some material components have a strong potential to induce inflammatory response and interfere with regulatory cellular networks (16, 48); thus, apart from additive inflammatory effects in established periodontal inflammation associated with oxidative stress (49, 50), resin monomers exposed to LPS may disrupt inflammatory networks (18, 48, 51) and further contribute to a less effective or detrimental immune response. From perspective of research studies aiming at development of new restorative materials, these findings suggest the importance of a meticulous assessment of periodontal inflammatory parameters in stage of pre-clinical and clinical phases to ascertain the safety of experimental materials, particularly in case of those with bioactive properties. In the spirit of immunomodulatory materials aiming at suppression of pro-inflammatory effects within biomaterial integration (52), the addition of anti-inflammatory components into restorative materials might be a promising research avenue.

Although the results demonstrate that carries and its treatment affect periodontal inflammatory and oxidative status, the present study has some limitations thus the future well-designed prospective studies in larger sample with longer follow-up are required to confirm the findings and to provide an in-depth knowledge about the biological effects of carries and its restorative materials on periodontium. The observed variability in baseline biomarker values between the groups also suggests the need for establishing the correlation between periodontal inflammatory and oxidative markers and extension of carious destruction. With this regard, the inclusion of untreated caries as positive control would certainly clarify this aspect as well; hence, although this would be incompatible with ethical regulations in humans, the future animal studies might address this important issue. Additionally, the controlled prospective studies on larger sample size are required to establish dynamics and nature of long-term biological response on caries and restorative materials over the time. Finally, experimental and clinical studies are also required to establish the nature of interactive effects between restorative materials and inflammatory patterns in state of periodontal disease.

Within limitations of the study, it is demonstrated that caries affects periodontal inflammatory and oxidative status, while its treatment appears to restore periodontal homeostasis. The standard temporary and restorative materials did not provide direct harmful effects on healthy periodontium, however the alteration of inflammatory and oxidative periodontal markers with material-specific patterning has been observed.

## Data Availability Statement

The original contributions presented in the study are included in the article/supplementary material. Further inquiries can be directed to the corresponding author.

## Ethics Statement

The studies involving human participants were reviewed and approved by Ethical Committee of the Military Medical Academy, Ministry of Defense, Serbia. The patients/participants provided their written informed consent to participate in this study.

## Author Contributions

Conceptualization: TK, MR, AS, and DV. Methodology: DV and MR. Formal analysis: AP-C and GS. Investigation: ET, VS, BD, and MD. Resources: MR. Data curation: ET, VS, BD, and MD. Writing—original draft preparation: MR, TK, AS, and DM. Writing—review and editing: MR and AS. Supervision: DM and BD. Project administration: DV. Funding acquisition: MR. Project administration: MR. Funding acquisition: MR. All authors have read and agreed to the published version of the manuscript. All authors contributed to the article and approved the submitted version.

## Funding

This work was funded by grants from the Serbian Ministry of Science and Technological Development, Grants No. ON175069, the Serbian bilateral project with PR China (06/2018), and the Faculty of Medical Sciences, University of Kragujevac (JP18/19and JP19/19) and supported by the Medical Military Academy, Republic of Serbia (MFVMA 07/22-24).

## Conflict of Interest

The authors declare that the research was conducted in the absence of any commercial or financial relationships that could be construed as a potential conflict of interest.

## Publisher’s Note

All claims expressed in this article are solely those of the authors and do not necessarily represent those of their affiliated organizations, or those of the publisher, the editors and the reviewers. Any product that may be evaluated in this article, or claim that may be made by its manufacturer, is not guaranteed or endorsed by the publisher.
